# The difficult management of disseminated *Sporothrix brasiliensis* in a patient with advanced AIDS

**DOI:** 10.1186/s12981-015-0051-1

**Published:** 2015-05-07

**Authors:** Ariane Gomes Paixão, Maria Clara Gutierrez Galhardo, Rodrigo Almeida-Paes, Estevão Portela Nunes, Marcelo Luiz Carvalho Gonçalves, Gisele Larias Chequer, Cristiane da Cruz Lamas

**Affiliations:** Fundação Oswaldo Cruz - Fiocruz, Universidade Grande Rio, Rio de Janeiro, RJ Brazil

**Keywords:** Sporotrichosis, AIDS, Amphotericin B, Posaconazole

## Abstract

Sporotrichosis is an infection caused by a dimorphic fungus of the *Sporothrix schenckii complex.* Host immunity is an important factor in the clinical manifestations of the disease. Deeply immunocompromised individuals, especially those infected with the Human Immunodeficiency Virus *(*HIV) and T CD4 counts < 350 cells/ul lymphocytes, may present with the systemic form of sporotrichosis. This report describes a case of disseminated sporotrichosis caused by *S. brasiliensis* in a patient with advanced AIDS. The skin, lungs, bones and central nervous system were affected. Medical treatment involved the administration of amphotericin B, terbinafine, itraconazole and posaconazole. Posaconazole was associated with the best clinical response and clearing of the fungus from the central nervous system.

**Electronic supplementary material:**

The online version of this article (doi:10.1186/s12981-015-0051-1) contains supplementary material, which is available to authorized users.

## Introduction

Sporotrichosis may be an acute or chronic infection caused by a dimorphic fungus of the *Sporothrix schenckii complex* [1] generally located in cutaneous and sub-cutaneous tissue, often associated with lymphatic involvement. The fungus can disseminate to bones, lungs and central nervous system, especially in immunosuppressed patients [2-6]. Recent publications have drawn attention to the possibility of dissemination related to fungal virulence factors, as is the case for *S. brasiliensis* [7].

The disease usually manifests as isolated cases or outbreaks related to specific occupational exposure; however, recently, it has reached epidemic proportions in some parts of Latin America, India [8] and Northeastern China [9]. The clinical, demographic and epidemiologic features vary within regions. The disease affects predominantly tropical and subtropical regions, being the most prevalent subcutaneous mycosis in South America. In Brazil, it is the most common cutaneous mycosis, and, in the Rio de Janeiro metropolitan area, it has been considered an epidemic zoonosis since the 1990s [2]. In this area, sporotrichosis occurs mostly via transmission from infected cats to humans, and genotypic analysis has shown that samples isolated from the Rio de Janeiro epidemic have a high genetic similarity. This finding is suggestive of a common niche, as *S. brasiliensis* is the species most frequently found in the city and in its surroundings [2,7]. Currently, this species is considered the most virulent of the *Sporothrix schenckii* complex [10]. On the other hand, the immune system of the host is an important factor that contributes to the different forms of the disease [11-14]. HIV co-infection clearly modifies the clinical consequences of the disease because T CD4 lymphocytes play an important role in the control of this mycosis. HIV infection aggravates sporotrichosis, with a higher incidence of severe disseminated cases and a higher number of hospitalizations and deaths [15]. The classical presentation of sporotrichosis is polymorphic lesions in the skin and sub-cutaneous tissue, compromising the adjoining lymphatics. The most affected cutaneous sites are those where the skin is most exposed to the environment. The incubation period is on average three weeks from the inoculation date.

The most frequent clinical form is the lymphocutaneous form, followed by the cutaneous form and the disseminated form. This report details a case of disseminated sporotrichosis in an HIV-positive female patient with advanced HIV immunosuppression.

### Clinical case

LSC, a 20- year- old female from Campos dos Goytacases (41°19’28”W), a city in the rural part of the state of Rio de Janeiro ( 274 km from Rio de Janeiro city), was diagnosed HIV positive in March 2011. She was a student and single. Due to the lack of laboratory resources in the patient’s hometown, CD4 and viral load were not collected at the time of diagnosis, and zidovudine (AZT), lamivudine, lopinavir and ritonavir were prescribed six months later due to progressive weight loss and fatigue.

On January 2012, the patient was scratched by her cat on the left hand, where the first polymorphic lesions (vegetative, nodular, ulcerous and crusty) appeared on the skin, spreading afterwards. Because of the severe skin condition, the patient was admitted on April 2012 to the Ferreira Machado Hospital in Campos dos Goytacases where she received deoxycholate amphotericin B (AMB) and itraconazole 600 mg/day, in addition to the antiretroviral therapy. A total dose dose of 2,05 g AMB was given along the 45 days of hospitalization. She showed no improvement in her condition and was therefore transferred to a referral Infectious Diseases hospital.

**Figure 1:**
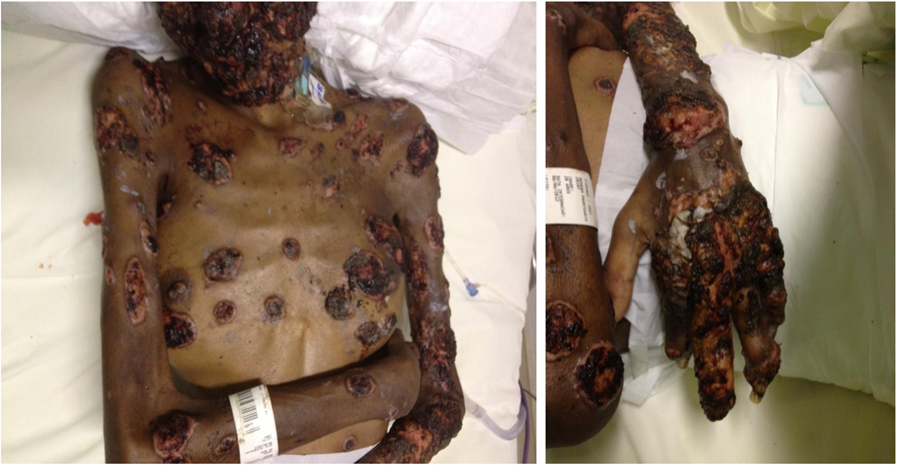
**Photographs on admission to IPEC/FIOCRUZ on June, 2012.** Polymorphic Sporotrichosis Lesions.

On June 2012, the patient was transferred to the Institute of Clinical Research (ICR) Evandro Chagas/FIOCRUZ (IPEC) due to the difficulty in managing the spreading polymorphic skin lesions associated with a wasting syndrome, severe protein-caloric malnutrition and the worsening of her general condition (Figure 1A, B).

**Figure 2:**
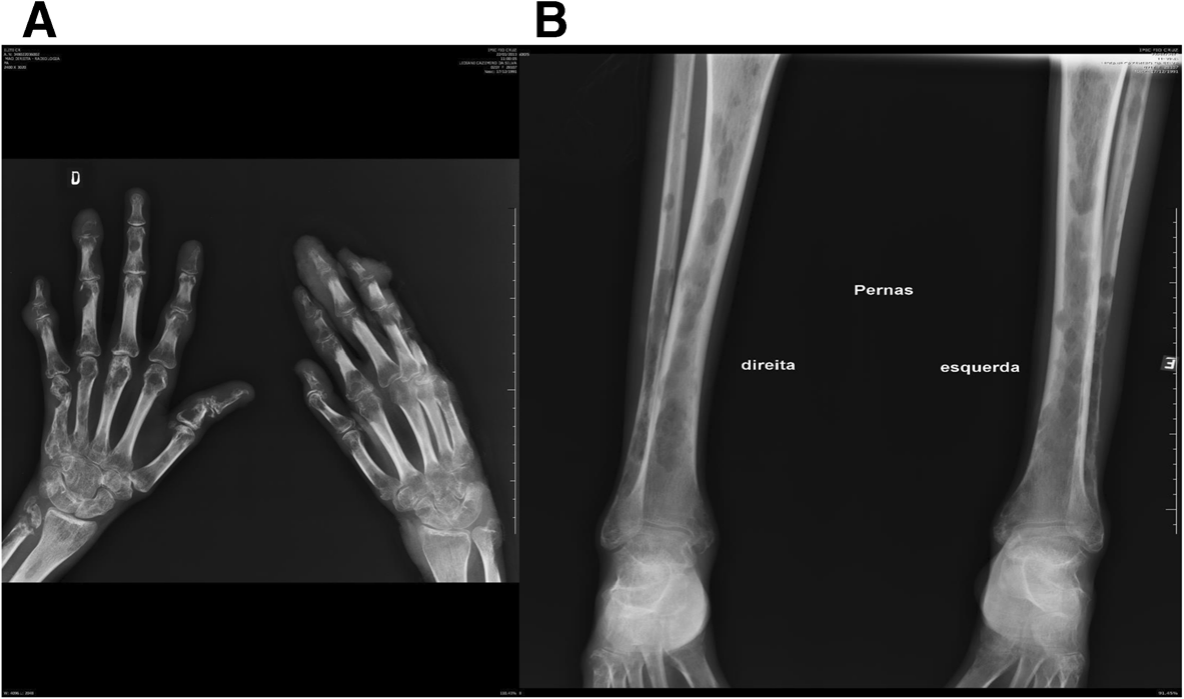
**Plain radiographs of hands (A) and lower limbs (B) showing osteolytic lesions (on June 2012, admission to IPEC).**

**Figure 3:**
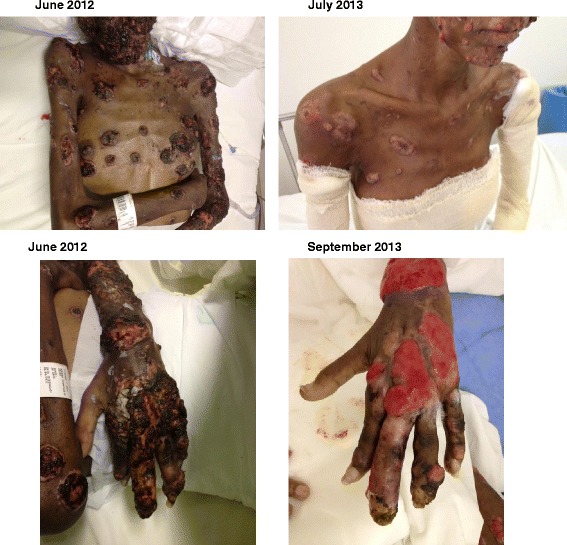
**Evolution of the skin lesions after treatment.** Evolution of the skin lesions.

Initial exams at the ICR showed macrocytic anemia (hemoglobin 10,3 g/dl; hematocrit 31,1%, MCV 105); leukocytosis 18110 mm3 with 10% band forms; C reactive protein of 32.48 mg/dl (reference level < 0.5), CD4 111 cells/mm3 (11,65%); CD8 605 cells/mm3 (63,75%); and a viral load of 92 copies/mm3. Cultures from blood, cerebrospinal fluid (CSF), induced sputum, urine and secretions from skin lesions were performed using Sabouraud, Mycosel and BHI-Agar and resulted positive for *Sporothrix spp.* A subsequent partial sequencing of the calmodulin gene showed that the isolate was actually *S. brasiliensis*. Plain radiographs and computed tomography (CT) scans showed osteolytic lesions, possibly due to fungal invasion. A bone biopsy was not performed because there was no local facility available for this type of procedure; moreover, given the results of all other exams, the biopsy was considered to be invasive and unnecessary (Figure 2A, B). At first, treatment with amphotericin B deoxycholate (1 mg/Kg/day; patient’s weight was 45 Kg) was maintained, and later on, it was substituted for a liposomal formulation. On July 16, 2012, treatment with itraconazole 200 mg/day was resumed and terbinafine 250 mg/day was associated. The patient had been taking zidovudine, lamivudine, lopinavir and ritonavir, but AZT was changed to abacavir due to severe anemia detected on August 2012. Skin lesions improved slowly and gradually, probably as a result of the antifungal and antiretroviral treatment and the weekly sessions of cryotherapy, which went on for 18 months (Figure 3A, B, C, D). The patient also presented an improvement in general condition with a weight gain of 6 Kg. She was able to stand and walk with assistance following weeks of intensive physiotherapy.

However, even with the general improvement and triple antifungal therapy with liposomal AMB, itraconazole and terbinafine, *S. brasiliensis* still grew in CSF that was collected up to October 2012, despite the fact the patient had no neurological symptoms. Because of the difficulty in sterilizing the CSF, on September 2012, terbinafine and itraconazole were stopped and posaconazole was prescribed at the dose of 800 mg/day [16,17], still associated with deoxycholate AMB. The administration of posaconazole was continuous but the AMB was not, as deep intravenous access was difficult and increases in serum creatinine occurred. The fungal susceptibility testing (Additional file 1) was done with the M38A2 method and CLSI 2008 standards on the *S. brasiliensis* isolated from the skin, sputum and CSF. The test results showed good sensitivity to terbinafine and voriconazole, intermediate sensitivity to AMB, and low sensitivity to itraconazole and ketoconazole. Posaconazole was not tested because there were no cut offs defined for this fungus; instead, its sensitivity was determined by comparing it to previous reports of *S. brasiliensis* isolates [18].

Mycological surveillance was performed monthly, and the first sterile CSF was seen on November 2012, a month after starting treatment with posaconazole. CSF sterility was documented until October 2013, despite the progressive cytological and biochemical changes seen in CSF (Additional file 1). The inflammatory changes led to the suspicion of meningeal tuberculosis or immune reconstitution syndrome, and empirical therapy with rifampin, isoniazid, pyrazinamide and ethambutol was given for 28 days and associated with dexamethasone (8 mg IV tds) for 14 days. At this point, the patient presented a CD4 cell count of 304 (11%) and a viral load < 40 copies/ml (Additional file 1).

**Figure 4:**
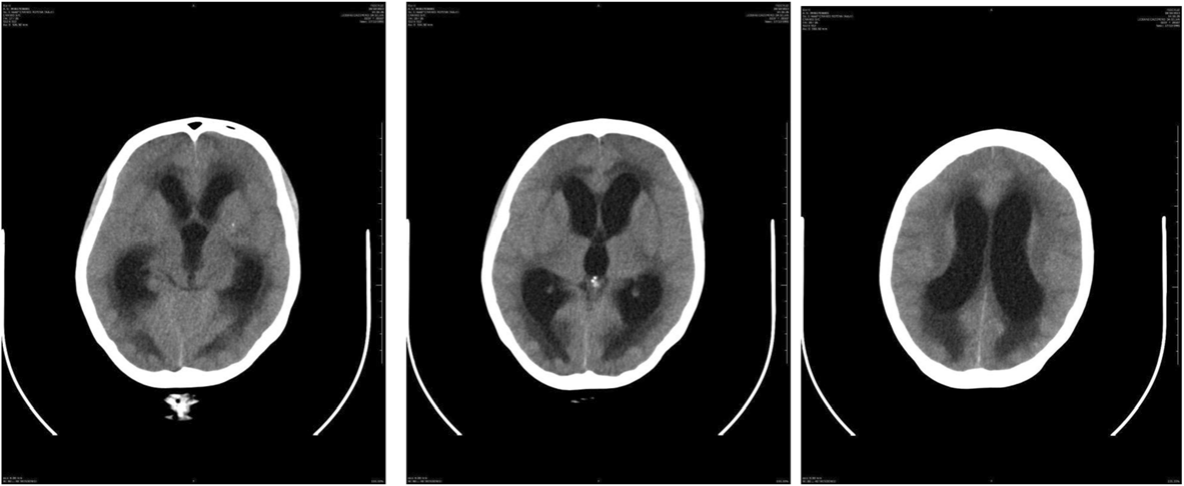
**Brain CT Scan on October 2013 showing exudative hydrocephalus.**

**Figure 5:**
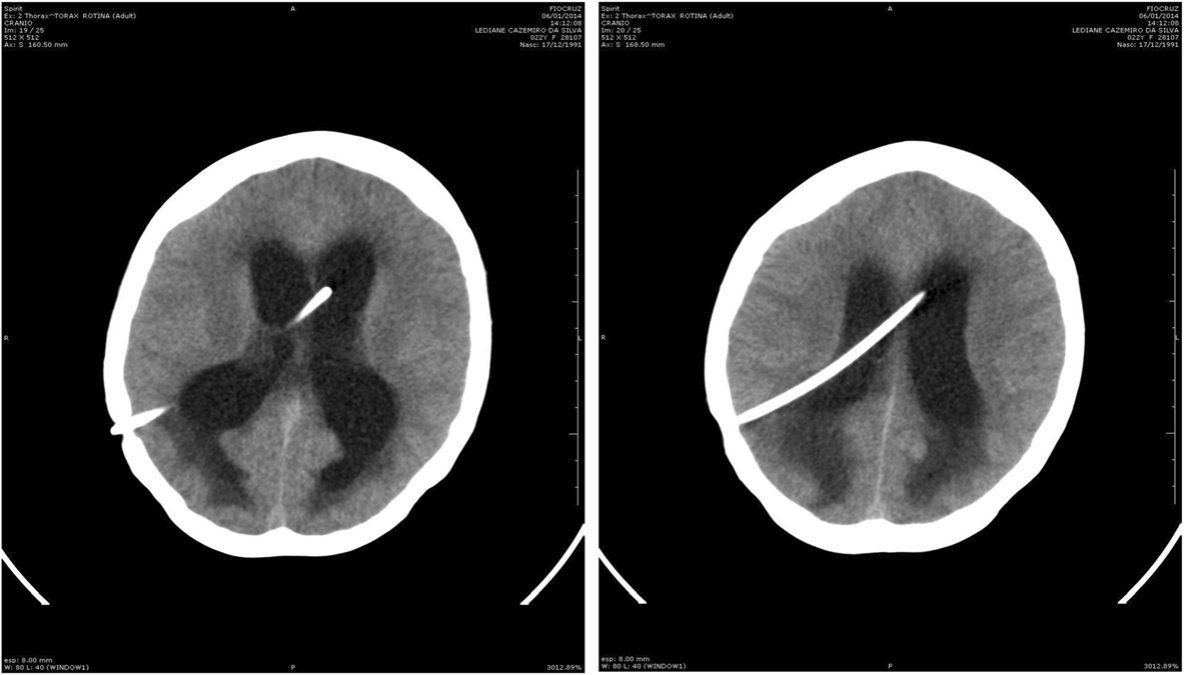
**Brain CT scan on January 2014, showing intraventricular shunt in place and hydrocephalus.**

**Figure 6:**
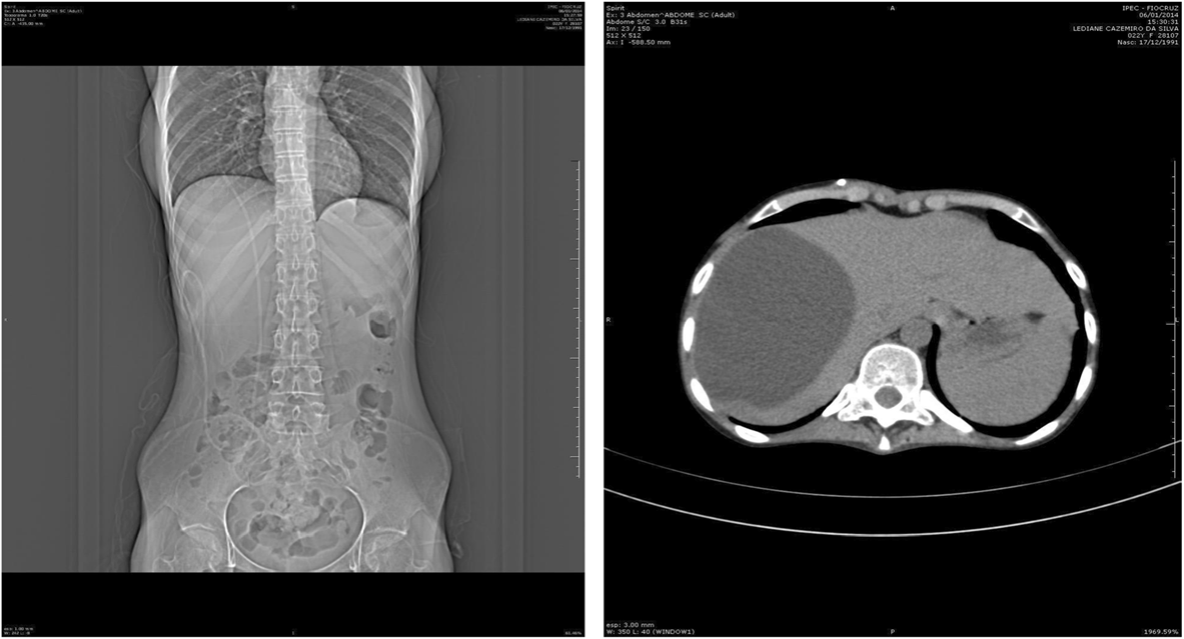
**Abdomen CT Scan, January 2014, showing infrahepatic fluid collection (CSF).**

During October 2013, the patient presented confusion, headache and nystagmus. Repeat brain CT scans showed exudative hydrocephalus, hypodensity of the white substance adjacent to the lateral ventricles, compatible with cerebrospinal fluid transudation, cortical sulci deletion and intense meningeal enhancement (Figure 4). Concurrently, CSF culture on November 4, 2013, was positive for *S. brasiliensis*. The patient was given a ventriculoperitoneal shunt (VPS) at the Bonsucesso General Hospital on October 22, 2013, with significant improvement of the neurological status. Approximately two months after placement of the CSF shunt, the patient once again presented with the same neurological symptoms and signs: CT scans showed recurrence of the hydrocephalus (Figure 5A, B) and an organized intrahepatic CSF collection, which indicated obstruction of the shunt (Figure 6A, B). On January 2014, the patient was given a new shunt replacement, and a few hours after the procedure, she passed away from an undetermined cause.

## Discussion

A severe case of *S. brasiliensis* disseminated sporotrichosis in a young female HIV-positive patient from Rio de Janeiro State in Brazil is presented. The severity of the case is shown by the isolation of the strain *S. brasiliensis* from cultures of the skin, sputum, blood and central nervous system, and the probable fungal osteomyelitis present radiologically. Virulence studies in a mouse infection model have shown that *S. brasiliensis* is significantly more lethal and results in higher fungal burdens compared to *S. schenckii* as well as other examined *Sporothrix* spp. Recently this species was also associated to aggressive and atypical cases [10,19].

Severity was also related to the poor clinical and mycological response to itraconazole and amphotericin B (the latter in high dose) during many months. Although itraconazole has been shown to treat *S. schenkii complex* adequately in a cohort of patients from the Rio de Janeiro metropolitan area [20], the strain isolated from our patient was itraconazole resistant. This finding raises the point that the itraconazole resistance found over time in strains in Rio de Janeiro could be related to the use of itraconazole to treat sick cats, which are the main source of *S. brasiliensis*. In this specific case, the patient had received itraconazole for 45 days at the time the first isolates were submitted for fungal sensitivity testing, which results showed itraconazole resistance.

The use of a combination of antifungals has not been described for treating sporotrichosis; however, for other mycosis such as chromoblastomycosis, this combination has been of clinical benefit [21]. *In vitro* synergy was observed with the combination of terbinafine and itraconazole against one isolate of *C. carrionii*, and no antagonism was observed when amphotericin B was combined with terbinafine or itraconazole against agents of chromoblastomycosis [21]. Weekly cryotherapy sessions occurred for a long period and resulted in the improvement of infiltrated cutaneous lesions, therefore favoring the skin penetration by antifungal agents.

Posaconazole is a modern azole and was chosen based on its good sensitivity profile to *S. schenckii (*and recently to *S. brasiliensis)* and its good penetration in bone and in the central nervous system [22,23]. Posaconazole showed both in *vitro* and in vivo action against a wide variety of fungi, including the rare and resistant ones. Several case reports describe success in the treatment of refractory fungal diseases or in patients who are unable to tolerate other drugs. Additionally, posaconazole can be given orally and is well tolerated [16]. Although the profile of voriconazole susceptibility in our isolates pointed us to a good sensitivity, based on previous studies that showed high MIC values in *S. schenckii*, associated with a lack of clinical studies, we decided not to use it, preferring posaconazole. In the present report, the use of posaconazole for two months coincided with fungal clearance from bi-monthly CSF cultures and a sustained response for 10 months.

It is not clear why the patient relapsed. It may be that posaconazole absorption was erratic, as has been suggested in other reports [24], and levels in CSF may have not been sufficient to suppress fungal growth. Alternatively, the isolate may have become resistant. These seem to be the most likely reasons because immune response had significantly improved at the time relapse occurred.

This case illustrates how serious sporotrichosis-HIV coinfection is and the need for frequent mycological surveillance, especially in cases where the central nervous system is involved. Also *S. brasiliensis* may present resistance to antifungals and is a virulent species.

## Conclusions

*S. brasiliensis* sporotrichosis and HIV coinfection is a serious condition. Treatment is an unresolved issue, but posaconazole seems to be an effective drug. There is need for frequent mycological surveillance, especially in cases where the central nervous system is involved. More studies are needed in order to confirm the benefit of posaconazole treatment in *S. brasiliensis* infections.

## Consent

Written informed consent was obtained from the patient for the publication of this report and any accompanying images.

## Additional file

Additional file 1: Table A. Profile of antifungal susceptibility using different strains of *S. brasiliensis*. **Table B.** Evolution of CSF patterns in the patient with Sporotrichosis and HIV. **Table C.** Results of cultures, CD4 cell count and treatment in a patient with disseminated sporotrichosis and HIV infection.

## Abbreviations

AMB: Amphotericin B
AZT: Zidovudine
CSF: Cerebrospinal fluid
CT: Computed tomograph
HIV: Human Immunodeficiency Virus
ICR: Institute of Clinical Research
VPS: ventriculoperitoneal shunt
