# Supplementary material for: The difficult management of disseminated Sporothrix brasiliensis in a patient with advanced AIDS
Source: AIDS Res Ther. 2015 May 7;12:16. doi: 10.1186/s12981-015-0051-1 (PMC4422263; doi:10.1186/s12981-015-0051-1)
Supplement: Additional file 1: Table A. — Profile of antifungal susceptibility using different strains of S. brasiliensis. Table B. Evolution of CSF patterns in the patient with Sporotrichosis and HIV. Table C. Results of cultures, CD4 cell count and treatment in a patient with disseminated sporotrichosis and HIV infection. [file 12981_2015_51_MOESM1_ESM.docx]

**Table A.**  Profile of antifungal susceptibility using different strains of *S. brasiliensis*

| Strain | Itraconazole | Voriconazole | Amphotericin B | ketoconazole | Terbinafine |
| --- | --- | --- | --- | --- | --- |
| 43987-1  (skin) 06/14/2012 | 2.0 | 2.0 | 2.0 | 0.5 | 0.03 |
| 43987-2  (skin) 06/14/2012 | 2.0 | 2.0 | 2.0 | 1.0 | 0.03 |
| 44022  (sputum) 06/20/2012 | 2.0 | 2.0 | 4.0 | 0.5 | 0.06 |
| 44262  (skin) 06/24/2012 | 2.0 | 4.0 | 4.0 | 1.0 | 0.06 |
| 43994  (CSF)* 06/15/2012 | 2.0 | 2.0 | 2.0 | 1.0 | 0.03 |

*Methods: M38A2 (CLSI 2008); * CSF=cerebrospinal fluid

The *S. brasiliensis* in this case presented good sensivity to terbinafine and voriconazole, medium sensitivity to amphotericin B and low sensitivity to itraconazole and ketoconazole, since the MIC for these drugs was above the necessary concentration to inhibit 50% of *S. brasiliensis* isolates in previous publications.

**Table B**. Evolution of CSF patterns in the patient with Sporotrichosis and HIV.

| Date of Lumbar  Puncture | Proteinorrhaquia  mg/dl | Glicorrhaquia  mg/dl | Cellularity  Number( cell type) | AFB * | VDRL | Gram  stain | Cultures |
| --- | --- | --- | --- | --- | --- | --- | --- |
| 06/15/2012 | 33.3 | 59 | 3(100%mono) | Neg | Non reag | Neg | *S. brasiliensis* |
| 10/09/2012 | 43 | 46 | 4(100%mono) | Neg | Non reag | Neg | *S. brasiliensis* |
| 11/22/2012 | 46.3 | 48 | 121(100%mono | Neg | Non reag | Neg | *Neg* |
| 04/24/2013 | 100.6 | 45 | 45(45%mono) | Neg | Non reag | Neg | *Neg* |
| 09/12/2013 | 311 | 29 | 68(80%mono) | Neg | Non reag | Neg | *Neg* |
| 10/13/2013 | 159 | 34 | 89(80%mono) | Neg | Non reag | Neg | *S. brasiliensis* |
| 12/12/2013 | 3936 | 24 | 17(90%mono) | Neg | Non reag | Neg | *Neg* |
| 01/06/2014 | 2047 | 23 | 2(100%mono) | Neg | Non reag | Neg | *Neg* |

Source: Exams collected at the IPEC/FIOCRUZ, AFB= acid fast bacilli; VDRL= veneral disease research laboratory; mono=mononuclear cells; neg=negative; non-reag=non reagent

**Table C**. Results of cultures, CD4 cell count and treatment in a patient with disseminated sporotrichosis and HIV infection.

| Dates | June/12 | Aug/12 | Oct/12 | Nov/12 | May/13 | Jul/13 | Oct/13 | Dec/13 | Jan/14 |
| --- | --- | --- | --- | --- | --- | --- | --- | --- | --- |
| CD4 cells/ul  (count; %) | 111; 11.6 |  |  | 94; 10.2 |  | 304; 11 |  |  | 192; 8.9 |
| Viral load( copies/ml) | 92 |  |  | 95 |  | <40 |  |  | 42 |
| **Fungal culltures: *Sporothrix* spp *^¶^*** | | | | | | | | | |
| Blood | Pos | Neg | Neg |  | Neg | Neg | Neg |  |  |
| Sputum | Pos |  | Neg |  |  | Neg | Neg |  |  |
| CSF | Pos | Pos | Pos | Neg | Neg | Neg | Pos | Neg | Neg |
| Skin lesion aspirate | Pos | Pos | Neg |  |  |  |  |  |  |
| Urine | Pos | Neg |  |  |  | Neg |  |  |  |
| **Antifungal therapy** | | | | | | | | | |
| AMB deoxycholate | X |  |  | X`` | X`` |  |  |  |  |
| AMB Liposomal |  | X |  |  |  |  |  |  |  |
| AMB Lipidic |  |  |  |  |  |  | X |  |  |
| Terbinafine 250mg/dia |  | X |  |  |  | X |  |  |  |
| Itraconazole200mg/dia |  | X |  |  |  | X |  |  |  |
| Posaconazole 800mg/dia |  |  | X | X | X | X | X | X | X |
|  |  |  |  |  |  |  |  |  |  |

Methods: Sabouraud, Micosel and BHI-Agar ; pos=positive, neg=negative; AMB=amphotericin B;

AMBdeoxycholateX = 2,05g total accumulated dose until June2012, X`:3.89g until November2012, X``: 5.15g until May2013;

AMB Liposomal X: 9.0g total accumulated dose until August 2012;

AMB Lipidic X = 4.05g total accumulated dose until October 2013.
